# The Effects of Social Presence on Adherence-Focused Guidance in Problematic Cannabis Users: Protocol for the CANreduce 2.0 Randomized Controlled Trial

**DOI:** 10.2196/resprot.9484

**Published:** 2018-01-31

**Authors:** Manuel Amann, Severin Haug, Andreas Wenger, Christian Baumgartner, David D Ebert, Thomas Berger, Lars Stark, Marc Walter, Michael P Schaub

**Affiliations:** ^1^ Swiss Research Institute for Public Health and Addiction University of Zurich Zurich Switzerland; ^2^ Clinical Psychology and Psychotherapy Friedrich-Alexander University Erlangen Nuremberg Erlangen Germany; ^3^ Department of Clinical Psychology and Psychotherapy University of Bern Bern Switzerland; ^4^ Arud Centre for Addiction Medicine Zurich Switzerland; ^5^ Division of Addictive Disorders University of Basel Psychiatric Clinics Basel Switzerland

**Keywords:** cannabis, mental disorders, adherence, social presence, mobile health, cognitive behavioral therapy

## Abstract

**Background:**

In European countries, including Switzerland, cannabis is the most commonly used illicit drug. Offering a Web-based self-help tool could potentially reach users who otherwise would not seek traditional help. However, such Web-based self-help tools often suffer from low adherence.

**Objective:**

Through adherence-focused guidance enhancements, the aim of this study was to increase adherence in cannabis users entering a Web-based self-help tool to reduce their cannabis use and, in this way, augment its effectiveness.

**Methods:**

This paper presents the protocol for a three-arm randomized controlled trial (RCT) to compare the effectiveness of (1) an adherence-focused, guidance-enhanced, Web-based self-help intervention with social presence; (2) an adherence-focused, guidance-enhanced, Web-based self-help intervention without social presence; and (3) a treatment-as-usual at reducing cannabis use in problematic users. The two active interventions, each spanning 6 weeks, consist of modules designed to reduce cannabis use and attenuate common mental disorder (CMD) symptoms, including depression, anxiety, and stress-related disorder symptoms based on the approaches of motivational interviewing and cognitive behavioral therapy. With a target sample size of 528, data will be collected at baseline, 6 weeks, and 3 months after baseline. The primary outcome measurement will be the number of days of cannabis use on the preceding 7 days. Secondary outcomes will include the quantity of cannabis used in standardized cannabis joints, the severity of cannabis dependence, changes in CMD symptoms, and adherence to the program. Data analysis will follow the intention-to-treat principle and employ (generalized) linear mixed models.

**Results:**

The project commenced in August 2016; recruitment is anticipated to end by December 2018. First results are expected to be submitted for publication in summer 2019.

**Conclusions:**

This study will provide detailed insights on if and how the effectiveness of a Web-based self-help intervention aiming to reduce cannabis use in frequent cannabis users can be improved by theory-driven, adherence-focused guidance enhancement.

**Trial Registration:**

International Standard Randomized Controlled Trial Number Registry: ISRCTN11086185; http://www.isrctn.com/ISRCTN11086185 (Archived by WebCite at http://www.webcitation.org/6wspbuQ1M)

## Introduction

### Epidemiology

According to the European Drug Report 2016 [1], approximately 13% of Europeans in the age range of 15 and 34 years have used cannabis in the last year; it is by far the most commonly used illicit drug. Translated into absolute numbers, there are approximately 16.6 million Europeans who use cannabis, with roughly one percent of European adults estimated to use cannabis daily [1]. Within Europe, Switzerland ranks third in the national prevalence of cannabis use: the lifetime prevalence rate of cannabis use is 27.9% (men 32.8%, women 23.2%), whereas the 6-month prevalence rate is 5.4% (men 7.6%, women 3.3%). The highest prevalence is found in the age group between 15 and 24 years. Within this group, the 6-month prevalence rate is 14.4%, with almost one-fifth of users using cannabis daily [2].

Even though these numbers are high, only a minority of cannabis users seem to develop cannabis dependence; in general population surveys, the risk of becoming dependent on cannabis appears to be between 10% and 11% among all cannabis users [3,4]. However, within the subgroup of cannabis users who started using cannabis in early life, the risks of cannabis dependence [5] and cannabis use problems [6] are much higher.

Although cannabis dependence has not been shown to increase mortality in the general population, its impact on the global burden of disease should not be understated. Expressed in disability-adjusted life years (DALYs), which is the sum of years of life lost because of premature mortality and years lived with disability, it has been estimated that, in 2010, cannabis dependence accounted globally for approximately two million DALYs [7].

Furthermore, poorer mental and physical health, lower educational achievement, and decreased cognitive functioning are all commonly seen in daily cannabis users [8]. Numerous studies also point to the associations between a broad range of primary mental illnesses and frequent cannabis use, highlighting the potential for detrimental effects of co-occurring mental health disorders on treatment in problematic cannabis users [9].

Patients who seek treatment for their cannabis use disorder at Swiss outpatient addiction treatment centers are mainly young males in the age range of 15 and 24 years [10]. Data from the monitoring system for addiction counseling and addiction treatment in Switzerland suggest a linear increase in new patients for whom cannabis is the main problem substance from 2004 (8.8%) to 2014 (46.7%) [11], a trend that has also been observed in other European countries [1]. However, it is clear that even though the number of treatment seekers has steadily increased, they still represent the minority of problematic cannabis users (eg, scoring 8 or higher on the Cannabis Use Disorder Identification Test) [12] who could potentially profit from treatment, among whom approximately half develop cannabis dependence [5], and many suffer from comorbid mental health problems [9].

### Accessibility and Reachability

There are various possible reasons why the percentage of problematic cannabis users who seek treatment is still so low. First among them are problems with accessibility: as addiction treatment centers are rare, low accessibility force potential treatment seekers, especially those living in more rural areas, to travel considerable distances. This increase in time required for travel is especially a hindrance, given that most addiction counseling centers operate during normal office hours, rendering attendance for potential treatment seekers almost impossible if they have a job that requires them to work during these hours [10,13]. The second reason pertains to the issue of stigmatization, with the fear of being stigmatized as a drug addict likely preventing many problematic cannabis users from seeking face-to-face treatment. As older individuals generally have greater social responsibilities, it is quite possible that the levels of fear increase as the age of users increase. Consistent with this assumption, users of Web-based self-help tools for the reduction of problematic cannabis use appear to be older and use more cannabis than those who seek help at outpatient addiction treatment centers [10].

Issues relating to self-efficacy also can be problematic, as some users have the desire to quit or reduce their cannabis consumption on their own [14]. Finally, many cannabis users lack insight into the various problems potentially caused by their cannabis use. For example, although many users are aware of some side effects of problematic cannabis use, such as mild depressive symptoms, the physical risks associated with the combustion and inhalation of smoke are often overlooked. These risks include heart disease, lung cancer, and chronic obstructive pulmonary disease [15,16]. Frequent users are more prone to disregard these risks than occasional users, but increased awareness could be advantageous for all, as it has been shown that the more users know about the possible physical risks of problematic cannabis use, the greater their intention to quit or at least reduce their cannabis use is [15,16].

Web-based self-help programs to reduce problematic substance use, such as—in the case of cannabis—CANreduce, could be a great asset in reaching those groups within the general population who would otherwise not seek treatment. Such programs facilitate access by being available around the clock and easily reachable from any home that has a computer and an Internet connection. Nowadays, many Web-based self-help programs also work on mobile devices, making almost any situation a potential treatment session. Apart from this huge improvement in accessibility relative to regular bricks and mortar treatment centers, Web-based programs also solve the fear of stigmatization issue. Allowing for anonymity, as well as enabling self-efficacy, it is often an ideal solution for at least a subgroup of users. Apart from all this, these noninvasive tools are low-cost and, depending on how they are set up (eg, whether or not they have counselors on standby for chat sessions), require little maintenance effort, lowering the costs once developed to just Web server space, domain name registration, minimal administrative support, and the effort needed to regularly update techniques and design. Naturally, this is of great interest amid the current environment of constantly increasing health care costs, as is the case in Switzerland and other industrialized countries [17].

### Previous Studies and Implications

However, all the aforementioned positive characteristics of such Web-based self-help programs only hold true if they actually are effective in their goal of reducing problematic cannabis consumption. This has indeed been consistently shown for programs aiming to reduce problematic alcohol use [18,19] and also—albeit to a lesser extent and not as consistently—for programs aiming for tobacco smoking cessation [20,21]. The Web-based approach for the most commonly used illicit drug, cannabis, has not been studied as thoroughly. To our knowledge, only two Web-based self-help programs to reduce problematic cannabis consumption have been tested for their effectiveness in appropriate RCTs in adults [10,22].

“Reduce Your Use: How to Break the Cannabis Habit” is an Australian self-help intervention that is fully automated and consists of six modules which are—similar to CANreduce—based on cognitive behavioral therapy (CBT), motivational interviewing (MI), and behavioral self-management. In an RCT, this intervention was shown to be more effective at reducing the frequency and quantity of cannabis consumption than a psychoeducational control condition. Regarding adherence to the 6-week program, two-thirds of the initial participants completed the intervention [22].

In addition, a previous version of CANreduce has been investigated with the objective of determining whether a Web-based self-help tool for reducing problematic cannabis consumption would be more effective if individualized chat sessions with a professional coach are offered [13]. Although the study found that participants who were in the group with the opportunity for individual chat sessions reduced their frequency of cannabis use more than those who only worked with the self-help tool, the effect was also identified among those in a chat sessions group who did not actually make use of the chat offer (ie, who did not reply to chat-based consultation appointments offered by professional chat counselors). Thus, it appears that even just the option of having chat appointments reduced cannabis consumption [10]. With only a quarter of participants who were given the option of scheduling a chat session actually taking advantage of the offer, the question was raised as to whether the same effect could be recreated by simpler means, eliminating the need for professional counselors on constant standby.

With CANreduce 2.0, we try to address this problem by implementing adherence-focused guidance, which has already been documented to be effective at increasing adherence to Web-based self-help relative to Web-based self-help alone [23,24]. The concept of adherence-focused guidance is primarily based on the supportive-accountability model of guidance in Web-based interventions [25] that asserts that unguided self-help programs are often less effective than those that are guided [26]. However, Mohr’s model for adherence to electronic health (eHealth) interventions encompasses further factors, which we additionally attempt to address, to strengthen the concepts of accountability and legitimacy with the assumption of thereby increasing the effectiveness of the support in terms of adherence. Specifically, we emphasize the *social presence* factor and those factors that increase program legitimacy as defined by Mohr’s model [25].

Furthermore, almost two-thirds of CANreduce participants in the former study screened positive for clinically relevant depression symptoms at baseline [10]. Moreover, even if partakers of intervention programs do not exhibit depressive symptoms at baseline, it is possible that such symptoms could emerge as they decrease their drug use. Comorbidity of depressive symptoms and substance use and its hindrance on positive treatment outcomes has been demonstrated several times before [27]. Furthermore, targeting a reduction in depressive symptoms in patients simultaneously could possibly increase adherence to the program. Therefore, the new version of CANreduce also aims to specifically target issues that potentially help to ameliorate depression and other common mental disorder (CMD) symptoms such as those associated with anxiety and stress-related disorder using CBT [27] and teaching social problem-solving skills [28]. Moreover, we incorporated aspects within modules targeting excessive rumination and worry, as well as difficulties with relaxing.

### Study Aims

The study presented in this protocol seeks to investigate and compare the effectiveness of Web-based self-help interventions at reducing cannabis use in problematic users, while taking into account the most frequently co-occurring mental health symptoms. Moreover, it examines whether adherence to the program can be optimized by emphasizing the social presence factor of adherence-focused guidance, which would only marginally increase costs versus guidance that is less personal.

## Methods

### Study Design

**Figure 1:**
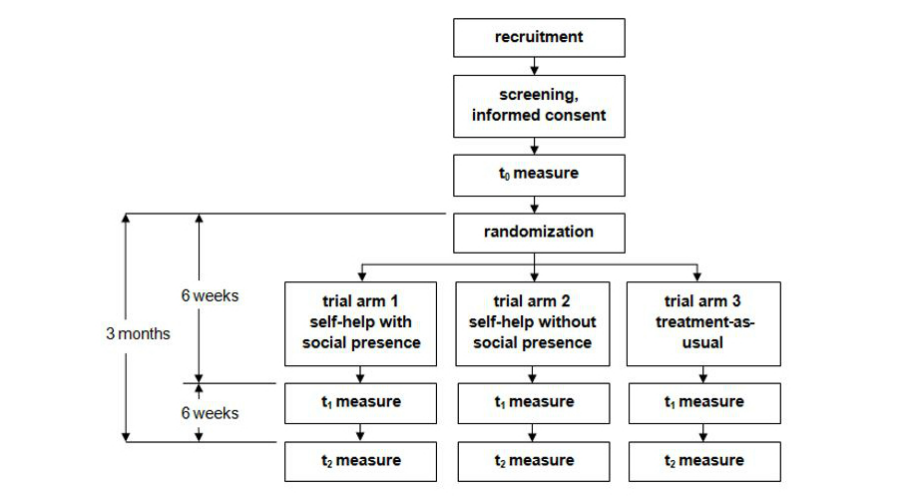
Trial flowchart.

The Web-based self-help program CANreduce will be evaluated within a three-arm RCT, comparing the effectiveness of an adherence-focused, guidance-enhanced, Web-based self-help intervention with a social presence; an adherence-focused, guidance-enhanced, Web-based self-help intervention without a social presence; and treatment-as-usual (TAU) at reducing cannabis use in problematic users. The masking technique will be partially single-blinded—in that participants in either of the two active treatment groups will not know which version they work with. The two versions are neutrally described as two differently optimized variants to prevent participants from having a preference of one over the other possibly resulting in a disappointment when being allocated to the unwished version. However, subjects will know whether they have been assigned to TAU. Any blinding of research and study personnel is not warranted. After successful completion of the baseline assessment (t0), participants will be allocated to one of the three study conditions. Further assessments will take place both 6 weeks (t1) and 3 months (t2) after baseline (Figure 1). The trial has been registered with the ISRCTN registry and is traceable as ISRCTN11086185.

### Recruitment of Study Participants

Recruitment will take place from August 2016 to December 2018 to ensure that the target sample size of 528 participants has been included. Participants will be recruited through the CANreduce website itself [29], which is already established and has links to it on various Internet health portals. Additionally, advertisements will be placed in relevant Internet forums and newspapers (or Web-based versions thereof).

For compensation, all participants who complete 3 months of follow-up from the start of the program will be able to choose between an Web-based voucher worth 30 Euro or donating that amount to charity.

### Registration and Consent Procedure

Participants can register online and will need to provide only minimal personal data, including their email addresses, phone numbers (only to get in contact with if follow-up questionnaires are not filled out), and some basic demographical data (age, gender, and level of education).

Before registration, each participant will be informed online about the study, specifically provided information about the following:

1. Purpose, background, and an overview of the study
2. Inclusion and exclusion criteria (Tables 1 and 2)
3. The difference between the three treatment arms (but no details about how the two active treatment arms differ) and their one in three chance of being allocated to each one of these three arms
4. Financial aspects (no participation fee, compensation for participation)
5. Potential risks of participation and when to contact their general practitioner or, alternatively, a professional they can select from a medical advisory and emergency list that will be accessible at all times merely by clicking on emergency icon
6. The inability of CANreduce to replace face-to-face therapy for problematic cannabis use or abuse
7. The voluntary nature of participation and their right to withdraw from the study at any time without consequences, except for the loss of further compensation
8. Confidentiality and data protection (anonymity is ensured by not recording real names or postal addresses and by deleting email addresses and phone numbers before statistical analysis and data archiving)
9. The approval given by the ethics committee of the Canton Zurich after the committee had reviewed the study

Informed consent will be accepted once participants have activated several check boxes restating important study points and have submitted their consent by clicking a submission button.

### Randomization and Trial Flow

Once participants have completed their baseline assessment, they will be randomized by a computer algorithm in a 1:1:1 ratio into one of three parallel groups, and this assignment will be registered automatically in the background database. 

**Table 1:** Inclusion criteria and underlying rationale.

Inclusion criteria	Reasoning
Informed consent via the Web form	To ensure knowledge of procedures and the declaration of consent
Minimal age of 18 years	To ensure a minimum age of participation
Cannabis use at least once weekly over the last 30 days	To include participants with less than daily cannabis use, increase validity
At least once weekly Internet access and a valid email address	To ensure at least some access to the intervention
Good command of the German language	To ensure that participants will be able to understand the information provided

**Table 2:** Exclusion criteria and underlying rationale.

Exclusion criteria	Reasoning
Participation in other psychosocial or pharmacological treatments for the reduction or cessation of cannabis use	To avoid confounding treatment effects
Current pharmacologically treated psychiatric disease or any history of psychosis, schizophrenia, bipolar type I disorder, or significant current suicidal or homicidal thoughts	To avoid having subjects with these problems enter the study

As participants assigned to TAU will immediately become aware of this, we expect that some might try to circumvent their assignment by registering another account, hoping to end up in a different group. If anyone attempts this, he or she nevertheless will be assigned to the original group when they try to register again on the same day based upon his or her Internet protocol address.

Participants will be introduced step by step into the corresponding study arm and, in the cases of study arms 1 and 2, be invited to participate in the program. Participants assigned to study arm 3 will be informed that they will be provided access to the Web-based self-help treatment after 3 months (with TAU until they are 3 months past their baseline assessment). Participants in study arms 1 and 2 will receive automated email notifications to log in and enter their cannabis consumption quantity and frequency into their consumption diary every week.

The two experimental interventions will each last 6 weeks. Follow-up assessments will be 6 weeks and 3 months after the start of the program. As such, there will be a baseline (pretreatment) assessment, a 6-week assessment immediately following the treatment program, and a final assessment 6 weeks posttreatment (3 months postbaseline).

The control condition will be TAU. As these subjects will have access to any other online and offline drug counseling services that are available, they will be asked about their possible use of other treatment services over the course of observation at their final follow-up visit.

Follow-up assessments should be completed online after a reminder is sent by email, in which they also will be reminded that, upon completion of the entire 3-month follow up assessment, they will be compensated with 30 Euro (via either an Web-based voucher or the choice to make an online charitable donation). If the final assessment is not completed within 2 days, the same reminder will be sent out two more times, 2 days apart. If these reminders still go unanswered, participants will be contacted by phone within 1 week after the third email has been sent and offered an interview on the phone with study collaborators to complete the follow-up instruments. Should participants still refuse, they will be asked to answer questions about the primary outcomes only or—should they still refuse—to provide a reason for refusing, which will then be documented.

It is not uncommon for participants of Web-based interventions to take breaks, yet still complete the intervention later. Therefore, there are no dropout criteria relating to inactivity. The only dropout criterion is active withdrawal from the study by the participant, in which case only the data already gathered will be analyzed.

Figure 1 shows a flow chart depicting the flow of subjects through the study.

### Hypotheses

We will test the following detailed study hypotheses with respect to the main outcome: reduction in the number of days of cannabis use over the past week, comparing the baseline, 6-week, and 3-month follow-up assessments:

1. An adherence-focused, guidance-enhanced, Web-based self-help program with a social presence (study arm 1) is more effective than an adherence-focused, guidance-enhanced, Web-based self-help program with no social presence (study arm 2) at reducing cannabis use.
2. An adherence-focused, guidance-enhanced, Web-based self-help program to reduce cannabis use with a social presence (study arm 1) is more effective than TAU (study arm 3) at reducing cannabis use.
3. An adherence-focused, guidance-enhanced, Web-based self-help program to reduce cannabis use without a social presence (study arm 2) is more effective than TAU (study arm 3) at reducing cannabis use.
4. Participants in study arm 1 will demonstrate better adherence than participants in study arm 2 over the 6-week intervention.

We have similar expectations with respect to our secondary cannabis-related outcomes and will explore frequently co-occurring mental disorders as predictors of adherence and outcomes. Specifically, we also want to explore, for the first time, the influence of attention deficit hyperactivity disorder (ADHD) symptoms on adherence to and outcomes from a Web-based intervention among problematic cannabis users.

### Intervention

CANreduce is an automated Web-based self-help tool developed by the Swiss Research Institute for Public Health and Addiction (ISGF) and and the Arud Centre for Addiction Medicine to reduce cannabis consumption in problematic cannabis users. The Web-based self-help intervention consists of a dashboard, a consumption diary, and eight modules designed to reduce cannabis use based on the principles of MI, self-control practices, and CBT methods. Participants can study all of the modules at their own pace and in whatever order they choose, though a specific order is advised.

As CANreduce 2.0 is regarded as a medical device because of the European Union guidelines 93/42/EWG and 2007/47/EWG, its conformity has been assessed, and potential risks have been evaluated. It is now fully certified in European conformity.

### Active Study Arms

The following elements of CANreduce will be used in both active treatment arms in this study (study arms 1 and 2). The social presence enhancements added just to the program offered to study arm 1 subjects will be described in the *social presence* section below.

#### Start Page

Before actual registration or log-in, a video is accessible in which the scientific director of the ISGF gives a quick introduction to CANreduce. This introduction can also be read as text and aims to motivate eligible individuals to participate in the study.

#### Dashboard

**Figure 2:**
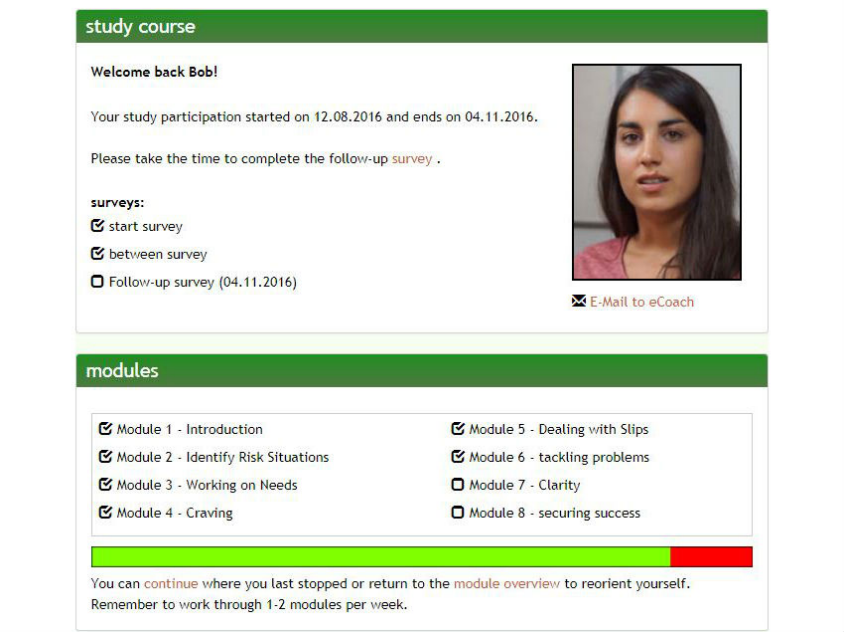
Dashboard for study arm 1 (translated from German to English for publication purposes only).

The dashboard (see Figure 2) serves as the central hub, displaying useful information at a quick glance. On the dashboard, participants can see the date when they started the program and how many days remain for them in it. It also displays the dates of the two follow-up assessments and indicates when they have been completed. The same is true of the individual intervention modules, and by clicking a link, participants will be taken to the page in the module where they left off the last time they logged in. There is also a way for subjects to directly enter cannabis consumption data over the preceding week, which then displays a consumption graph.

**Figure 3:**
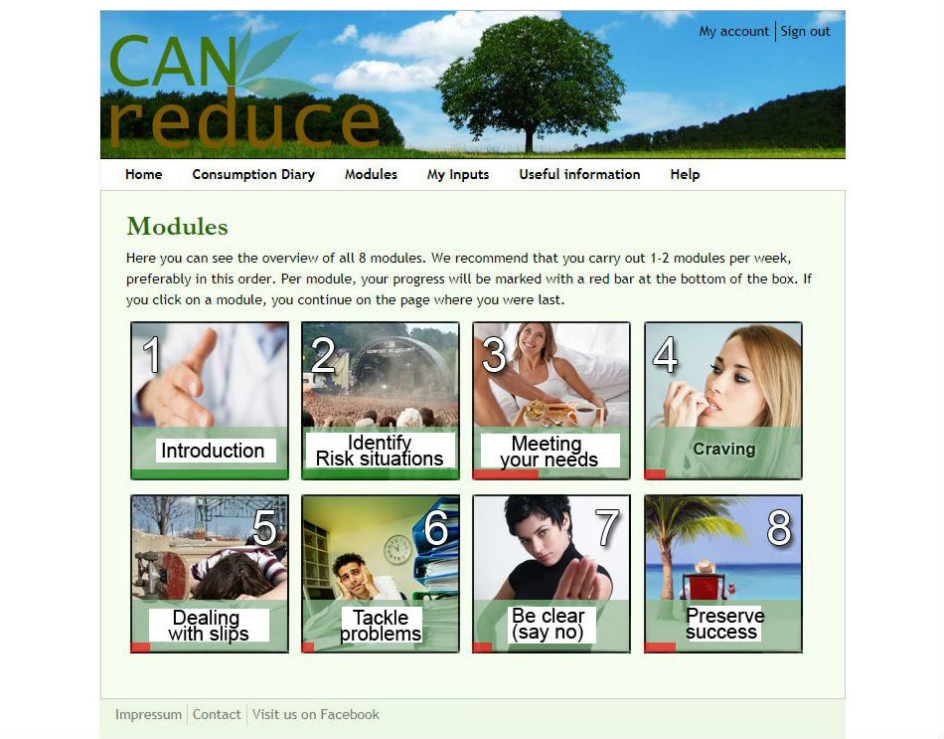
Main menu (translated from German to English for publication purposes only).

#### Self-Help Intervention Modules

There are eight self-help intervention modules that are the same for both active treatment groups in terms of the information presented. They are depicted on intervention website’s main menu page (see Figure 3), as well as on the dashboard. Participants are encouraged to complete either one or two of these modules each week and to complete all of the modules in order, though they can access all modules right away. It is also stated that they should feel free to jump directly to modules they feel could be of importance at the moment and to repeat any modules they feel they either especially need to take a second look at, or that they perceive to be especially helpful within the 6 weeks of the program.

A bar in the module overview will indicate the progress they have made with each module; that bar is fully green when the entire module is completed.

The eight modules are summarized in Table 3 and are described below.

##### Module 1: Introduction

In this module, which is largely based on MI techniques, a general overview of the program is given, and fictional companions are introduced. Additionally, participants are encouraged to state their personal reasons for and against their cannabis consumption, which they can review at any time, so they may reflect on what they could gain by successfully completing the program.

##### Module 2: Identifying Risky Situations

Apart from identifying personal situations in which participants could find it difficult to stand by their set consumption goals and working through these scenarios so they are better prepared when they arise, another focus of this module is on seemingly irrelevant decisions and chains of events that can potentially result in unplanned cannabis use. This is an area also explored in traditional CBT.

##### Module 3: Working on Needs

In this module, participants learn skills to help them strengthen their social contacts, decrease possible ruminations, and develop healthier sleeping habits. The importance of sleep and its impact on quality of life is explained. Participants are encouraged to install some rules or rituals to improve their sleeping quality (eg, no big meals or sports in the evening and taking time to unwind the day). Rumination and its impact on well-being is explained, and several techniques are presented to counteract such behavior (eg, thought stop, an audio file with a *passing clouds meditation*, or refocusing on positive things we are grateful for). Finally, social contacts and their link with mood are explained. Participants are encouraged to list people they wish to be in closer contact with, what they could do about it, and what negative beliefs inhibit them from doing so.

##### Module 4: Craving

Here, the concept of craving is explained with its physical and mental aspects for which participants are encouraged to state examples from their own experience. The key concept of triggers (conditioned stimuli) is explained and its link to risk situations, making module 2 worth a suggested revisit. Five possible ways to deal with craving are presented: distraction, talking about craving, mindful experiencing of craving, envisioning negative consequences of consumption, and self-talk.

##### Module 5: Dealing With Relapses

Temporary relapses can happen, but they should not decrease a person’s motivation to achieve their personal consumption goal or be cause for grievance. In this module, participants are taught skills for relapse prevention and to not see relapses as catastrophic events, but rather to learn from them and view their consumption goals as a long-term process.

##### Module 6: Working on Problems

The relationships between cannabis consumption, problems in life, and mild depressive symptoms are highlighted in module 6. Here, participants learn how to deal with problems that they cannot personally affect and are shown problem-solving skills to help them to deal with problems that are seemingly too large to tackle.

##### Module 7: Saying “No”; Refusal Skills

In this module, different ways to strengthen refusal skills are taught to decrease the person’s risk of a relapse whenever and wherever they find themselves in high-risk situations.

##### Module 8: Preserving Achievements

In the eighth and final module, participants are encouraged to review their work in the program and then generate a list of five personalized points that can help them to secure their achievements once their participation in the program is completed.

#### Fictional Companions

Six fictional companions appear within the modules at key points, with the goal of encouraging reflection on certain questions raised by the modules. This is done by the companions thinking out loud, sharing their thoughts on issues raised in written form. Participants can choose one character that best fits their own situation but can also click through the input provided by the other five companions.

#### Consumption Diary

Participants are encouraged to fill out their cannabis consumption diary completely on at least a weekly basis. They can enter their goal about how much they want to use in upcoming days and how much they actually did use in past days in terms of the number of individually standardized joints. 

This individual joint is defined at the start of the program where participants can select between (1) indoor, outdoor, or resin; (2) mixed with or without tobacco; and (3) six different amounts of the substance varying from 67 mg to 500 mg depicted with six photos of unrolled joints corresponding to the type selections from (1) and (2).

**Table 3:** Overview of contents and therapeutic approaches in the modules.

Module	Contents	Therapeutic approach
Module 1: Introduction	General overview Introduction of fictional companions Reflection on personal cannabis consumption	Based on motivational interviewing (MI) techniques [30]
Module 2 : Identifying risk situations	Identifying personal high-risk situations Recognizing seemingly irrelevant but triggering decisions	Cognitive behavioral therapy (CBT) approach to relapse prevention [31]
Module 3: Working on needs	Strengthening social contacts Decreasing excessive ruminations Developing healthier sleeping habits	Behavioral activation approach [32]
Module 4: Craving	Concept of craving Ways to deal with feelings of craving	Based on CBT [33]
Module 5: Dealing with relapses	Relapse prevention Dealing with relapses	CBT approach to relapse prevention [31]
Module 6 : Working on problems	Relationships between consumption, problems, and depressive symptoms Skills to deal with solvable and unsolvable problems	Social problem-solving approach [34]
Module 7: Saying "no"; refusal skills	Strengthening refusal skills for use in high-risk situations	Based on CBT [33]
Module 8: Preserving achievements	Review of program List of five personalized points to help secure achievements after the program is complete	Based on MI techniques [30]

Overall, there are 36 different photos made for this selection procedure [10,13]. A graph is generated live with these data inputs and provides the participant with visual feedback. The ability to anonymously set daily consumption goals could possibly counteract the self-deception often seen in face-to-face drug counseling and strengthen the self-efficacy of users. By adding this consumption diary to both active study arms, Mohr’s accountability factor—*goal setting*—is also integrated into the program.

#### Other Elements

The CANreduce tool further consists of a section with general useful information regarding cannabis, such as physical risks and harm reduction techniques. Furthermore, in some modules, participants are asked to enter their answers to certain exercises (either by clicking on checkboxes or entering text freely). These data are accessible in the section *My Contribution*.

#### Automated Motivational Email Feedback

Each week, participants in intervention arms 1 and 2 will be sent automated motivational email feedback that will contain a reminder to fill out their consumption diary and a direct link to the CANreduce log-in site. If participants do not fill out their diary, they will receive a different reminder email 1 and 3 days later.

Additional emails will be sent out, either automatically or triggered by an administrator or moderator, if certain conditions are met—for example, if an increase in a participant’s cannabis consumption or stalling of consumption reduction is detected—as well as encouraging emails to work with the self-help tool if only a few modules have been completed after 2 and 4 weeks. These feedback emails will also include module suggestions, depending on how subjects have responded in exercises dealing with high-risk situations, cravings, or the pros and cons of their consumption.

In the first CANreduce study [10], participants sometimes discontinued their use of the Web-based self-help tool simply because they had initially aimed for a minimal reduction in consumption and reached this self-set goal within the first few weeks. Therefore, depending on their goals entered into the consumption diary, participants will receive a message encouraging them to reduce their consumption by at least 20 percent over the first week and, if they succeed, to continue that trend in subsequent weeks. Alternatively, participants who do not succeed at reaching their goal will receive the suggestion that they aim for a more modest goal and continue until their final consumption goal is reached.

Another finding from the first version of CANreduce was that some users seek not only to reduce their consumption, but to become completely abstinent. When this goal was achieved early in the program, some felt less inclined to continue with the program. This will be addressed in this study with an automated email, triggered when actual consumption is recorded as zero for several days consecutively in a subject’s consumption diary, encouraging them to nonetheless complete certain modules of importance to their current situation (eg, preventing and dealing with relapses).

One important component of adherence-focused guidance, as defined by Ebert et al [24], is adherence monitoring. Through these mostly automated emails, either encouraging participants to work with the self-help tool or suggesting modules that they have not yet completed, this will be implemented in both active study arms. Another crucial element of adherence-focused guidance is feedback on demand. This too has been implemented in both active study arms by inviting participants at the end of each email to send in any questions that might arise for them during the course of the program. For subjects in the first study arm, this option will also be displayed on the dashboard. Although the content of these nonautomated answers is not specific to either study arm, the format of the emails will differ between study arm 1 and 2, as described next, under *social presence*.

#### Social Presence

Certain specific enhancements have been implemented in study arm 1 in accordance with points made in Mohr’s supportive accountability model [25]. Added social presence aims to give the self-help tool a personal feel, creating a form of alliance between the user and the Web-based self-help tool by replicating that aspect of human support. We aim to recreate rapport similar to what typically is found in client-counselor relationships, without the need to have such a counselor on constant standby. It has been argued by Mohr et al [25] that adherence increases when there is accountability to a coach, who should be seen as trustworthy, benevolent, and having expertise. By introducing a mostly automated eCoach named Deborah, we seek to quite literally give our Web-based tool a face. Although difficult to define one individual who implies the aforementioned attributes to all potential users of the self-help tool, we sought out an eCoach who appears visually friendly and supplemented the representation of benevolence with video scripts and emailed texts that suggest expertise, as well as trustworthiness (see Figure 2).

We created short introduction videos for most of the modules, in which Deborah greets the user, gives her opinion on the importance of certain key points within the module, and finishes by wishing the user an enjoyable time working on the module and acknowledging that they will see each other again in the next module. Additionally, a picture of the eCoach Deborah will be constantly displayed on the dashboard.

She also will personally invite users to write to her should they have any questions or problems. As in the chat version of the first CANreduce study [10], we expect that the number of participants who will actually take her up on this offer will be relatively small. However, we also expect that the assurance that they have the option to write to her will exert a positive impact on their adherence and, thereby, increase their treatment success. Emails written to the eCoach’s address will be answered by available study collaborators—if needed after consultation with a certified therapist; consequently, the answer that querying subjects will receive will address whatever issue or issues they have. In this way, however, the task of replying to emails can be spread out among multiple persons, while retaining that element of personal, one-on-one rapport. It is assumed that the content of this feedback will not, in itself, enhance the effectiveness of the program, but that having the option for direct contact with their own coach will enhance subjects’ perception of the coach’s benevolence and legitimacy.

Furthermore, automatic email reminders or module suggestions will have a greater personal touch in the adherence-focused, guidance-enhanced version with a social presence (Deborah) than in the version without the social presence (no Deborah). In such correspondence, the participants will be addressed by their username; emails and other written communication from the eCoach will be signed as “your eCoach Deborah”; and all text will be written from a first-person perspective (eg, modules will be recommended by her personally; see Textbox 1).

For technical reasons, only one eCoach has been introduced in this newer version of CANreduce. If the demand for personal feedback by the eCoach proves to be greater than expected, replies will be written by multiple coaches. As it could be perceived as deceitful if they all represented themselves as Deborah, the coaches will each sign those emails as an associate or representative of eCoach Deborah.

Therefore, although participants in both active study arms will receive the same level of support, those in the second study arm will be supported by an undisclosed entity, whereas the form of support provided to those in the first study arm will appear to be more intimate and more closely resembling face-to-face coaching.

#### Control Conditions

The control condition will be TAU, as it cannot be ruled out that participants who are allocated to the third study arm will seek out other treatment options during the waiting period. At the last follow-up assessment, participants will be asked if, over the course of their 3 months in the study, they used other treatments and what they were; these data will then be analyzed. After 3 months of follow-up, these subjects’ study phase will be finished, at which time they will be offered the opportunity to start the self-help program if they so choose.

A second control condition pertains to the presence versus absence of a social presence. As described above, there will be two active control groups, one with and one without the eCoach Deborah in their version of the self-help tool.

#### Technical Specifications

CANreduce 2.0 is based on the content management system Drupal 7, with a MySQL database. It will be administered internally by the information technology (IT) developer at the institution where the principle investigator (PI) works as faculty. All access will be administered via encrypted and password-protected secure sockets layer connections to canreduce.ch [29]. At any time, any participant will have access to his or her own data only. The administrator will have full access to all of the data from all subjects.

All data will be entered online by the users themselves. The website ensures a certain degree of data validation, such as accepting only numbers for number fields, ensuring that users do not mark multiple choices for single choice questions, insisting that certain mandatory fields are filled out, and so on.

**Module suggestion on the basis of open text answers for pros and cons for the keywords difficulty with falling asleep, tiredness, social inhibition, feelings of guilt, blaming oneself, and rumination.**

With social presence:

Hello [participant’s user name]!

You have been using the program for some time now and have already completed a few of the modules. Well done!

If I may, I’d like to make a recommendation. I looked at your pros and cons of using cannabis and want to point out module 3 “Working on needs.“

This module covers three important topics: better sleep, less rumination, and social contacts. Perhaps one of these topics will be particularly helpful in your current situation.

Why don’t you have a look at it this evening?

For questions or difficulties, feel free to contact me.

Best regards,

Your eCoach, Deborah

Without social presence:

Hello,

You have been using the program for some time now and have already completed a few of the modules. Well done!

On the basis of your pros and cons of using cannabis, module 3 “Working on needs” would be recommendable.

This module covers three important topics: better sleep, less rumination, and social contacts. Perhaps one of the topics would be particularly helpful in your current situation.

Why don’t you have a look at it this evening?

For questions, you can write to canreduce@canreduce.ch.

**Table 4:** Assessment instruments.

Assessment instruments	Initial assessment (t_0_)	Week 6 (t_1_)	3-month follow-up (t_2_)
Sociodemographics	X		
Center for Epidemiologic Studies Depression Scale	X		X
Short Screening Scale for DSM-IV^a^ posttraumatic stress disorder	X		X
General anxiety disorder-7	X		X
Adult ADHD^b^ Self-Report Scale-version 1.1	X		X
Quantity of cannabis use	X	X	X
Frequency of cannabis use	X	X	X
Cannabis Use Disorder Identification Test-Revised	X	X	X
Severity of Dependence Scale	X	X	X
Fragebogen Substanzanamnese	X	X	X
Client Satisfaction Questionnaire-I		X	
Intervention adherence^c^		X	
WAI-TECH^d^		X	
Negative effects according to Rozental			X

^a^DSM-IV: Diagnostic and Statistical Manual of Mental Disorders, 4th edition.

^b^ADHD: attention deficit hyperactivity disorder.

^c^Continuous assessment during 6 weeks.

^d^WAI-TECH: Working Alliance Inventory adapted for Web-based interventions.

CANreduce also features a responsive design and can automatically adapt to small screen devices such as tablets and mobile phones. Users can register online by choosing a username and providing a valid email address. To complete the registration process, users will have to click on a verification link sent to the email address specified, which will allow them to create a personal password, while preventing anyone from registering without a valid email address.

All data will be stored on a Web space hosted by an external provider that meets the IT security outsourcing regulations (99/2) of the Swiss Federal Banking Commission. Employees of the Web host will need to identify themselves with biometric data to gain physical access to the infrastructure.

Data will be extracted from the running Web-based database via Drupal and PHPmyAdmin. The data will then be stored at the PI’s institution on local computers for further processing and local file servers for archiving.

Each subject’s email address and phone number will be deleted after their participation in the study is complete and thereby, not available for either current or future analysis.

The investigator affirms and upholds the principle of every participant's right to privacy and that all personnel involved in the study will comply with applicable privacy laws. No individual data will ever be published or presented at scientific meetings.

### Measurements

Table 4 provides an overview of measurements. Sociodemographic data will include subject gender, age, and level of education.

The primary outcome of interest will be the number of days of cannabis use on the preceding 7 days according to the Time-Line-Follow-Back method [35,36].

Secondary outcomes of interest will include the quantity of cannabis used in the previous week, in standardized cannabis joints (as indicated in the consumption diary as well, as per Schaub et al [10]). Participants can choose between three different cannabis forms presented in photographs and in the second step between five different standard joints for each category (1/10 g, 1/6 g, 1/4 g, 1/3 g, and 1/2 g content pictures). These joints are either pure cannabis or cannabis mixed with tobacco. Finally, every participant has his or her personal standard tobacco cigarette, a ruler with centimeter and millimeter scales, the fraction amount in grams, and an open 10 cm paper prepared to roll a joint and containing the cannabis plant- or resin-tobacco mixture or pure cannabis presented in his or her consumption diary. Further secondary outcomes are the presence and severity of a cannabis use disorder (Cannabis Use Disorder Identification Test-Revised, CUDIT-R); the severity of cannabis dependence (Severity of Dependence Scale, SDS); the use of alcohol, tobacco, or other illicit drugs besides cannabis (Fragebogen Substanzanamnese, FDA); changes in depression, anxiety, and attention deficit symptoms (Center for Epidemiologic Studies Depression Scale, CES-D; general anxiety disorder-7, GAD-7; and Adult ADHD Self-Report Scale-V1.1, ASRS-V1.1); the Short Screening Scale for Diagnostic and Statistical Manual of Mental Disorders, 4th edition (DSM-IV) posttraumatic stress disorder (PTSD); client satisfaction (Client Satisfaction Questionnaire adapted to Internet-based interventions, CSQ-I); and treatment adherence. The perceived relationship between the user and the eCoach will be assessed at the end of the program using the Working Alliance Inventory [37] adapted for Web-based interventions (WAI-TECH).

The CUDIT-R is a questionnaire containing eight items designed to identify problematic cannabis consumption. Each item is a statement regarding cannabis use, to which respondents are provided with five response options, numbered from 0 to 4, that vary between questions, but for which increasing scores indicate increasing use or cannabis-related problems. As such, possible scores range from a minimum of 0 to a maximum of 32. A score of 8 or more indicates hazardous cannabis use, whereas a score of 12 or more indicates a possible cannabis use disorder [12].

The SDS is a reliable and valid 5-item screening scale, with a score of 4 or more being indicative of cannabis dependence [38].

The FDA asks about a person’s years of lifetime consumption, the past month’s consumption, and the manner of consumption for the DSM-IV or the International Statistical Classification of Diseases and Related Health Problems, 10th revision substances of abuse. This measure was derived from the EuropeASI, the European version of the Addiction Severity Index [39].

The CES-D Scale is a short self-report scale designed to measure depression symptoms in the general population [40]. All items on the scale are symptoms associated with depression that have been used in previously validated longer scales. CES-D responses rate the frequency at which depression symptoms have occurred over the past week. Possible scores on the CES-D-20 range between 0 and 60, where a CES-D-20 cutoff score of 16 is considered indicative of *significant* or *mild* depression symptoms. This is equivalent to experiencing six symptoms for most of the previous week or a majority of symptoms on 1 or 2 days. Higher scores indicate a higher symptom load.

The GAD-7 is a 7-item, self-report questionnaire to screen for and estimate the severity of GAD and has good reliability as well as factorial and concurrent validity [41]. These items ask about nervousness, inability to stop worrying, excessive worry, restlessness, difficulty relaxing, easy irritation, and the fear of something awful happening. Total scores range from 0 to 21, with a recommended cutoff score of 10 or higher.

The six-item short version of the ASRS-V1.1 can be self-administered easily and quickly [42]. With a total possible score of 24 and a cutoff score of 14, this six-item version has been shown to have strong concordance with clinician diagnoses, while significantly shorter than the full 18-item version.

The short screening scale for DSM-IV PTSD is designed to assess for a lifetime history of PTSD [43]. A score of 4 or more on the seven-symptom screening scale suggests PTSD.

The CSQ-I has been shown to be a suitable measure from the user’s perspective in the evaluation of Web-based health interventions. It is scored easily by summing up the individual item scores to produce a score ranging from 8 to 32, with higher scores indicating greater satisfaction [44].

Furthermore, the occurrence of any negative effects will be identified, as in Rozental et al, at the 3-month follow-up assessment [45].

Finally, we will ask all participants if they had used any other treatment than canreduce.ch during the 3 months and if so, to select from a predefined list of services.

As an indicator of treatment adherence, data will be collected on which modules have been completed by the participant and the number of weeks the consumption diary was filled out. Additionally, a script has been implemented that allows us to measure the time that subjects spend on each page. To avoid false data that might result if a participant leaves a Web page open but switches to a different window or leaves the computer, a cutoff time of 10 min inactivity has been set, after which the time spent on the page is disregarded and not saved to the database. These data could potentially lead to insights into how and specifically where to optimize CANreduce to further decrease attrition rates. We also will assess the number of individual emails received by each participant.

### Sample Size Calculation

Anticipating that a Cohen *d* of 0.30 based on our previous study experiences will be realistic for the effect size differences between the unenhanced version of the Web-based tool and the adherence-focused guidance-enhanced version, a sample size of n=176 in each study group would have 80% power to detect this difference based on calculations performed with G*Power software (Faul, Kiel) with an alpha error of 5% and two-tailed testing. Thus, we aim to recruit a total of 528 participants.

### Data Analyses

Data will be analyzed according to the intention-to-treat principle (ITT). To address missing data for the ITT analyses, we will apply multiple imputation procedures with the package Amelia in R (R Foundation for Statistical Computing, Vienna). Amelia uses a bootstrapping-based algorithm that gives essentially the same answers as the standard imputation posterior or expectation maximum approaches according to the authors. We plan to use between 20 and 40 imputed datasets depending on the amount of missing data according to suggestions by Graham [46]. The imputation model will include all primary and secondary outcome variables. Auxiliary variables such as demographic data may be included if they improve convergence of the imputation model.

Differences between study arms in primary and secondary continuous outcome variables at baseline and the follow-up points will be tested using linear mixed models (LMM). LMMs will be specified appropriately to model clusters and repeated measures by defining random effects for study arms and time (repeated measures). For nonnormal continuous outcomes, appropriate distributions (eg, negative binomial and zero-inflated) will be specified. For binary outcomes, a generalized linear mixed model (GLMM) will be specified that defines an appropriate link-function. In the GLMM fixed effect, coefficients will be interpreted in the context of the subject-specific (nonmarginal) model fit.

### Safety

Potential risks are expected to be minimal as no drugs will be administered and the medical device (ie, the self-help tool) was determined to be of very low risk during the course of its European conformity certification. What we expect to observe is some mild withdrawal symptoms such as craving, mild depressive states, and sleep problems. These issues will be addressed in the psycho-educational modules that are part of the 6-week self-help intervention. At all times, an *instant help* Web page will be available with instructions on what subjects can do if their situation becomes unbearable. These instructions contain psycho-educational self-help instructions, as well as phone numbers to professional health care providers.

## Results

The study will be conducted in accordance with the ethics board–approved protocol and the principles stated in the current version of the Declaration of Helsinki; the CONSORT eHealth Guidelines [47] for studies on medical devices; the European Directive on medical devices 93/42/EEC; and the ISO Norm 14155 and ISO 14971 as well as Swiss Law and Swiss Regulatory Authority requirements. The local ethics committee and regulatory authorities will receive annual safety and interim reports and be informed about study termination, in agreement with local requirements.

The study was approved by the ethics committee of the Canton of Zurich on July 4, 2016 (BASEC-Nr. 2016-00264) and is registered at Current Controlled Trials, traceable as ISRCTN11086185.

Results will be published in a scientific peer-reviewed journal. Anonymized study data will be available on request. Participants will be informed via email about study results via a lay-person-friendly summary of trial findings, if they have requested so at registration.

## Discussion

### Principal Findings

To the best of our knowledge, this study will be the first to assess the effectiveness—in terms of increasing adherence and treatment success—of adding a social presence to adherence-focused guidance by implementing a human element, in accordance with the supportive accountability model [25], all within the context of a Web-based self-help intervention program to reduce problematic cannabis consumption. The results of this RCT could add valuable insights into how to increase adherence to Web-based self-help tools, for which high attrition rates are one of the greatest reported problems. If users do not adhere to the created content, treatment success is unlikely. If these relatively simple techniques are shown to increase adherence, these ideas could be extrapolated further and easily applied to both current and future Web-based self-help tools to aid in reducing problematic substance use of any kind.

Although it must be noted that participants in the first CANreduce study who received at least one chat session still performed better than those in the same treatment condition who did not [10], the offer of being able to contact an eCoach who is perceived as benevolent, trustworthy, and having expertise by email, with any questions or problems, might adequately reproduce this beneficial effect.

Although adherence and treatment success have already been shown to be greater in interventions in which participants have the option of speaking with a therapist [10], the complexity and costs are increased as well, as such sessions need to be scheduled, and therapists must be available. Such increases in cost and complexity should not be a problem with the human element that we will be implementing, as it is a one-time effort to shoot the videos and prewrite emails with a personal feel. We expect that these features alone will increase program effectiveness; and furthermore, that very few actual emails will be sent by subjects seeking answers. If more emails than anticipated need to be answered individually, this task could be handled by multiple people to address less complex issues. Meanwhile, the email format itself should leave enough time for us to gather input from experts to address more complex questions.

To explore the possible effects of the added content addressing CMDs, we will compare outcomes against the study that examined the first version of CANreduce. Another avenue for exploration will be measuring adherence with specific modules that address CMDs.

### Limitations

The following protocol limitations must be considered:

First, cannabis users who are currently receiving other treatments to reduce their cannabis consumption will be excluded. However, CANreduce was designed to access cannabis users who—for personal or practical reasons—would not attend traditional addiction counseling. Second, all measurements will be self-reported. Third, most of the self-report instruments we will be using have not been validated in a Web-based context, though they have largely been validated in other research and clinical settings. Fourth, as found in the previous Web-based intervention [10,22], we expect rather high rates of dropouts. Finally, another possible limitation of Web-based studies is the potential for low adherence rate because of the distant nature of intervention.

### Conclusions

This study will evaluate the effectiveness of an enhanced version of CANreduce, a Web-based self-help intervention to reduce problematic cannabis consumption. If shown effective, the importance of CANreduce as a tool by which to reach users in the general population who otherwise would not seek out traditional mental health care, and addiction counseling services will be documented.

The benefits of the intervention include providing cannabis users with a better understanding of their addictive behaviors, teaching them psychological tools to handle drug cravings and prevent relapses, and, ultimately, helping them to become free of cannabis dependence.

Furthermore, if adding a social presence to adherence-focused guidance augments the program’s effectiveness, valuable insights could be gained into how to more effectively design Web-based interventions. These findings could then be adapted to other Web-based self-help tools, which present poor adherence and high attrition rates.

## Abbreviations

ADHD: attention deficit hyperactivity disorder
ASRS-V1.1: ADHD Self-Report Scale-V1.1
CBT: cognitive behavioral therapy
CES-D: Centre of Epidemiologic Studies of Depression Scale
CMD: common mental disorder
CSQ-I: Client Satisfaction Questionnaire adapted to Internet-based interventions
CUDIT-R: Cannabis Use Disorders Identification Test-Revised
DALY: disability-adjusted life year
DSM-IV: Diagnostic and Statistical Manual of Mental Disorders, 4th edition
eHealth: electronic health
FDA: Fragebogen Substanzanamnese
GAD-7: Generalized Anxiety Disorder-7
GLMM: generalized linear mixed model
ISGF: Swiss Research Institute for Public Health and Addiction
IT: information technology
ITT: intention-to-treat
LMM: linear mixed models
MI: motivational interviewing
PI: principle investigator
PTSD: posttraumatic stress disorder
RCT: randomized controlled trial
SDS: Severity of Dependence Scale
TAU: treatment-as-usual

## References

[1] (2016). European Monitoring Centre for Drugs and Drug Addiction.

[2] Marmet S, Notari L, Gmel C (2015). http://www.suchtmonitoring.ch/docs/library/marmet_bowy2kw8gwlc.pdf.

[3] Wagner FA, Anthony JC (2002). From first drug use to drug dependence: developmental periods of risk for dependence upon marijuana, cocaine, and alcohol. Neuropsychopharmacology.

[4] Degenhardt L, Hall W, Lynskey M (2001). The relationship between cannabis use and other substance use in the general population. Drug Alcohol Depend.

[5] Anthony JC, Roffman RA, Stephens RS (2006). The epidemiology of cannabis dependence. Cannabis Dependence: Its Nature, Consequences, Treatment.

[6] Fischer R, Clair C, Studer J, Cornuz J, Gmel G (2013). Prevalence and factors associated with use of smokeless tobacco in young Swiss men. Eur J Public Health.

[7] Degenhardt L, Ferrari AJ, Calabria B, Hall WD, Norman RE, McGrath J, Flaxman AD, Engell RE, Freedman GD, Whiteford HA, Vos T (2013). The global epidemiology and contribution of cannabis use and dependence to the global burden of disease: results from the GBD 2010 study. PLoS ONE.

[8] Fischer B, Jeffries V, Hall W, Room R, Goldner E, Rehm J (2011). Lower Risk cannabis use Guidelines for Canada (LRCUG): a narrative review of evidencerecommendations. Can J Public Health.

[9] Lev-Ran S, Le Foll B, McKenzie K, George TP, Rehm J (2013). Cannabis use and cannabis use disorders among individuals with mental illness. Compr Psychiatry.

[10] Schaub MP, Wenger A, Berg O, Beck T, Stark L, Buehler E, Haug S (2015). A web-based self-help intervention with and without chat counseling to reduce cannabis use in problematic cannabis users: three-arm randomized controlled trial. J Med Internet Res.

[11] (2016). Suchtschweiz.

[12] Annaheim B, Scotto TJ, Gmel G (2010). Revising the Cannabis Use Disorders Identification Test (CUDIT) by means of Item Response Theory. Int J Methods Psychiatr Res.

[13] Schaub MP, Haug S, Wenger A, Berg O, Sullivan R, Beck T, Stark L (2013). Can reduce--the effects of chat-counseling and web-based self-help, web-based self-help alone and a waiting list control program on cannabis use in problematic cannabis users: a randomized controlled trial. BMC Psychiatry.

[14] Ellingstad TP, Sobell LC, Sobell MB, Eickleberry L, Golden CJ (2006). Self-change: a pathway to cannabis abuse resolution. Addict Behav.

[15] Fernández-Artamendi S, Fernández-Hermida JR, García-Fernández G, Secades-Villa R, García-Rodríguez O (2013). Motivation for change and barriers to treatment among young cannabis users. Eur Addict Res.

[16] Becker J, Hungerbuehler I, Berg O, Szamrovicz M, Haubensack A, Kormann A, Schaub MP (2013). Development of an integrative cessation program for co-smokers of cigarettes and cannabis: demand analysis, program description, and acceptability. Subst Abuse Treat Prev Policy.

[17] Curry SJ (2007). eHealth research and healthcare delivery beyond intervention effectiveness. Am J Prev Med.

[18] Riper H, Spek V, Boon B, Conijn B, Kramer J, Martin-Abello K, Smit F (2011). Effectiveness of E-self-help interventions for curbing adult problem drinking: a meta-analysis. J Med Internet Res.

[19] Rooke S, Thorsteinsson E, Karpin A, Copeland J, Allsop D (2010). Computer-delivered interventions for alcohol and tobacco use: a meta-analysis. Addiction.

[20] Civljak M, Sheikh A, Stead LF, Car J (2010). Internet-based interventions for smoking cessation. Cochrane Database Syst Rev.

[21] Haug S, Schaub M (2011). Wirksamkeit internetbasierter Programme zum Tabakrauchen [in German]. Zeitschrift für Gesundheitspsychologie.

[22] Rooke S, Copeland J, Norberg M, Hine D, McCambridge J (2013). Effectiveness of a self-guided web-based cannabis treatment program: randomized controlled trial. J Med Internet Res.

[23] Zarski A, Lehr D, Berking M, Riper H, Cuijpers P, Ebert DD (2016). Adherence to internet-based mobile-supported stress management: a pooled analysis of individual participant data from three randomized controlled trials. J Med Internet Res.

[24] Ebert DD, Lehr D, Heber E, Riper H, Cuijpers P, Berking M (2016). Internet- and mobile-based stress management for employees with adherence-focused guidance: efficacy and mechanism of change. Scand J Work Environ Health.

[25] Mohr DC, Cuijpers P, Lehman K (2011). Supportive accountability: a model for providing human support to enhance adherence to eHealth interventions. J Med Internet Res.

[26] Baumeister H, Reichler L, Munzinger M, Lin J (2014). The impact of guidance on Internet-based mental health interventions — A systematic review. Internet Interv.

[27] Hides L, Samet S, Lubman DI (2010). Cognitive behaviour therapy (CBT) for the treatment of co-occurring depression and substance use: current evidence and directions for future research. Drug Alcohol Rev.

[28] Platt JJ, Husband SD (1993). An overview of problem-solving and social skills approaches in substance abuse treatment. Psychotherapy.

[29] CANreduce.

[30] Miller WR, Rollnick S (1991). Motivational interviewing: Preparing people to change addictive behavior.

[31] Marlatt GA, Donovan D (2005). Relapse prevention: Maintenance strategies in the treatment of addictive behaviors.

[32] Kanter JW, Puspitasari AJ, Santos MM, Nagy GA (2012). Behavioural activation: history, evidence and promise. Br J Psychiatry.

[33] Steinberg KL, Roffman R, Carroll K, McRee B, Babor T, Miller M, Davidson C (2005). Brief Counseling for Marijuana Dependence: A Manual for Treating Adults. Rockville, MD: Center for Substance Abuse Treatment, Substance Abuse and Mental Health Services Administration.

[34] Jaffee WB, D'Zurilla TJ (2009). Personality, problem solving, and adolescent substance use. Behav Ther.

[35] Hjorthøj CR, Hjorthøj AR, Nordentoft M (2012). Validity of Timeline Follow-Back for self-reported use of cannabis and other illicit substances--systematic review and meta-analysis. Addict Behav.

[36] Robinson SM, Sobell LC, Sobell MB, Leo GI (2014). Reliability of the Timeline Followback for cocaine, cannabis, and cigarette use. Psychol Addict Behav.

[37] Kiluk BD, Serafini K, Frankforter T, Nich C, Carroll KM (2014). Only connect: the working alliance in computer-based cognitive behavioral therapy. Behav Res Ther.

[38] Gossop M, Darke S, Griffiths P, Hando J, Powis B, Hall W, Strang J (1995). The Severity of Dependence Scale (SDS): psychometric properties of the SDS in English and Australian samples of heroin, cocaine and amphetamine users. Addiction.

[39] Kokkevi A, Hartgers C (1995). EuropASI: European Adaptation of a Multidimensional Assessment Instrument for Drug and Alcohol Dependence. Eur Addict Res.

[40] Cole JC, Rabin AS, Smith TL, Kaufman AS (2004). Development and validation of a Rasch-derived CES-D short form. Psychol Assess.

[41] Löwe B, Decker O, Müller S, Brähler E, Schellberg D, Herzog W, Herzberg PY (2008). Validation and standardization of the Generalized Anxiety Disorder Screener (GAD-7) in the general population. Med Care.

[42] Daigre C, Ramos-Quiroga J, Valero S (2009). Adult ADHD self-report scale (ASRS-V1.1) symptom checklist in patients with substance use disorders. Actas Esp Psiquiatr.

[43] Breslau N, Peterson EL, Kessler RC, Schultz LR (1999). Short screening scale for DSM-IV posttraumatic stress disorder. Am J Psychiatry.

[44] Boß L, Lehr D, Reis D, Vis C, Riper H, Berking M, Ebert DD (2016). Reliability and validity of assessing user satisfaction with web-based health interventions. J Med Internet Res.

[45] Rozental A, Boettcher J, Andersson G, Schmidt B, Carlbring P (2015). Negative effects of internet interventions: a qualitative content analysis of patients' experiences with treatments delivered online. Cogn Behav Ther.

[46] Graham JW, Olchowski AE, Gilreath TD (2007). How many imputations are really needed? Some practical clarifications of multiple imputation theory. Prev Sci.

[47] Eysenbach G, CONSORT-EHEALTH Group (2011). CONSORT-EHEALTH: improving and standardizing evaluation reports of Web-based and mobile health interventions. J Med Internet Res.

